# Highly trabeculated structure of the human endocardium underlies asymmetrical response to low-energy monophasic shocks

**DOI:** 10.1063/1.4999609

**Published:** 2017-08-24

**Authors:** Adam Connolly, Matthew D. Robson, Jürgen Schneider, Rebecca Burton, Gernot Plank, Martin J. Bishop

**Affiliations:** 1 Department of Biomedical Engineering, Division of Imaging Sciences and Biomedical Engineering, King's College London, London, United Kingdom; 2 Division of Cardiovascular Medicine, University of Oxford, Oxford, United Kingdom; 3 Division of Biomedical Imaging, University of Leeds, Leeds, United Kingdom; 4 Pharmacology Department, University of Oxford, Oxford, United Kingdom; 5 Institute of Biophysics, Medical University of Graz, Graz, Austria

## Abstract

Novel low-energy defibrillation therapies are thought to be driven by virtual-electrodes (VEs), due to the interaction of applied monophasic electric shocks with fine-scale anatomical structures within the heart. Significant inter-species differences in the cardiac (micro)-anatomy exist, however, particularly with respect to the degree of endocardial trabeculations, which may underlie important differences in response to low-energy defibrillation protocols. Understanding the interaction of monophasic electric fields with the specific human micro-anatomy is therefore imperative in facilitating the translation and optimisation of these promising experimental therapies to the clinic. In this study, we sought to investigate how electric fields from implanted devices interact with the highly trabeculated human endocardial surface to better understand shock success in order to help optimise future clinical protocols. A bi-ventricular human computational model was constructed from high resolution (350 *μ*m) *ex-vivo* MR data, including anatomically accurate endocardial structures. Monophasic shocks were applied between a basal right ventricular catheter and an exterior ground. Shocks of varying strengths were applied with both anodal [positive right ventricle (RV) electrode] and cathodal (negative RV electrode) polarities at different states of tissue refractoriness and during induced arrhythmias. Anodal shocks induced isolated positive VEs at the distal side of “detached” trabeculations, which rapidly spread into hyperpolarised tissue on the surrounding endocardial surfaces following the shock. Anodal shocks thus depolarised more tissue 10 ms after the shock than cathodal shocks where the propagation of activation from VEs induced on the proximal side of “detached” trabeculations was prevented due to refractory endocardium. Anodal shocks increased arrhythmia complexity more than cathodal shocks during failed anti-arrhythmia shocks. In conclusion, multiple detached trabeculations in the human ventricle interact with anodal stimuli to induce multiple secondary sources from VEs, facilitating more rapid shock-induced ventricular excitation compared to cathodal shocks. Such a mechanism may help explain inter-species differences in response to shocks and help to develop novel defibrillation strategies.

In this work, a high-resolution, anatomically faithful, computational model of the human ventricles is used to investigate the response of the human ventricles to monophasic electric shocks. A specific mode of shock-induced excitation is revealed which relies on the presence of fine-scale endocardial structures (specifically detached trabeculae), and does not occur symmetrically with respect to the shock-polarity. It is thought that this excitation mode may be important to consider when optimising low-energy defibrillation protocols, and also may contribute to the previously observed differences in response to shocks of different polarity. As the human endocardial anatomy is known to be significantly more trabeculated than other more commonly studied species, this mode of shock-induced excitation, and asymmetry in response with respect to shock-polarity, may be especially pronounced in humans.

## I. INTRODUCTION

Defibrillation via a strong applied electric field remains an effective, but far from ideal, therapy against episodes of otherwise lethal arrhythmias. At-risk patients often receive an implanted cardioverter defibrillator (ICD) that automatically applies such strong shocks via electrodes implanted across the heart when required. Current research is investigating the potential use of effective low-energy therapies,^1–7^ driven by the increasing concern of patients and clinicians on the use of strong shocks which limit battery-life, are an independent predictor of mortality,^8^ and present a significant burden of pain and resulting psychological issues due to inappropriate shocks.

Recent studies have suggested that low-energy shocks, applied via a specific protocol, can defibrillate with similar success rates to currently used single high-energy shocks.^1,2,4–7^ However, in contrast to the *biphasic* shocks currently used in ICDs, the proposed low-energy protocols involve a series of *monophasic* shocks, sometimes being followed by pacing stimuli.^5^ The specific voltages applied, numbers of stimuli, and relative timings differ between recent studies which have been performed on different species (canine^5–7^ and rabbit^2–4^); moreover, the specific choice of monophasic protocol and the reasons behind it are often not clear, as is the apparent preference for the use of the anodal shock polarity. These reasons suggest a lack of comprehensive understanding of the way in which monophasic electric fields interact with cardiac tissue, and potentially important interspecies differences between these responses.

The bidomain model of cardiac electrophysiology has helped explain how heterogeneities or discontinuities in the intracellular domain create “virtual-electrode” (VE)—regions of positive/negative polarisation induced remote from the physical electrodes between which the stimulus is applied.^9,10^ Bidomain theory says that current must traverse these heterogeneities in order to pass between the electrodes; as it does so it must either exit or enter the intracellular domain (through the membrane), causing either a depolarising or a hyperpolarising effect on the tissue, respectively. Bidomain theory has been used to explain the formation of VEs at tissue surfaces (epi-/endocardium), as well as intracellular structures such as blood vessels^6,7,11,12^ or around regions with varying fibre directions.^13^

Recent modelling and experimental studies have highlighted the importance of fine-scale anatomical structures^2,6,7,11,12,14^ in forming VEs during applied shocks, which may facilitate arrhythmia termination. Blood vessels have been suggested to be key in this regard,^6,7,11,12^ driving the formation of secondary sources of activation from the induced VEs that may help activate the bulk of the myocardium and more efficiently close excitable gaps. Such a mechanism has been proposed to underlie some of the recent novel low-energy defibrillation protocols,^6,7^ as well as facilitate biphasic defibrillation,^11,12^ emphasising their importance in inclusion in computational models.

Trabeculation structures on the endocardial surface have also been shown to elicit VEs,^2^ suggesting the need for their inclusion in detailed anatomical models. Trabeculae grooves or invaginations have been shown to provide sources of locally elevated membrane responses,^2,15,16^ due to confinement of electric field-lines,^16^ locally raising field strength as current takes the path of least resistance outside the myocardium (driven by differences in conductivity between tissue and blood in the cavity). The stronger VEs occurring in such grooves, relative to smooth surfaces, cause them to preferentially act as secondary sources of activation following shock-end, which again have been shown to be of relevance in low-energy defibrillation protocols.^2^ It has also been shown that positive and negative VEs can form on either side of isolated structures (such as papillary muscles, or detached trabeculations)^17,18^ as current enters/leaves the tissue, forming in close proximity for relatively small structures.

There exists a known significant difference in the endocardial structural detail between species, with humans known to have a highly trabeculated endocardium in comparison with other commonly used experimental preparations.^19^ We suggest that these anatomical differences in the trabeculae structure drive important differences in the response of the human ventricular myocardium to relatively weak electric fields, which may be relevant for understanding and improving low-energy defibrillation protocols in the clinic.

In this study, we investigate this hypothesis by the creation of an anatomically detailed model of the human ventricles, generated from high-resolution *ex-vivo* MR data, containing unprecedented levels of structural details. Shocks of different strength, but generally below the threshold for surface capture, are applied by simulated implanted electrodes, at different stages of tissue refractoriness. A novel mechanism for activation of the endocardium is uncovered, driven by VE formation around detached trabeculae, occurring specifically for anodal shocks. We show how this mechanism invokes a different response when applied to arrhythmia episodes, relative to cathodal shocks, which has important implications in understanding low-energy protocol optimisation in humans.

## II. METHODS

### A. Computational ventricular model generation

#### 1. MRI data

Experiments conformed to rules and regulations of the UK Human Tissue Authority (approved by the Oxford Research Ethics Committee). Fresh intact hearts (*n* = 1) were provided by the Oxford Heart Valve Bank (John Radcliffe Hospital Oxford, UK) for swift whole-heart scans, followed by return to the Valve Bank. Fresh intact hearts were Langendorff-perfused with Hartmann's solution (Baxter, UK) and scanned *in toto*. Whole hearts were scanned in a 120 mm (inner diameter) MRI specimen holder. Imaging was carried out on a 3 T (123 MHz, 120 mm tube; TimTrio, Siemens Medical Systems, Germany) MR system. Resolution was optimised according to the available scan time (as samples had to be returned for further processing by the Valve Tissue Bank), yielding 350 *μ*m isotropic resolution (in 12 min) in this case. The total dataset comprised some 384 × 384 × 251 voxels. The MR data were then cropped in the short-axis direction to remove the atria. Due to the specific orientation within the scanner, this resulted in removing part of the basal ventricles to ensure a planar clipping-plane in the axial direction; however, the majority of the ventricles were maintained, importantly including fine-scale endocardial structures closer to the apex. Unfortunately, a small section of the apex was also removed due to the bias-field artifact.

#### 2. Image processing and mesh generation

Image segmentation was performed based-on the methodology described previously^11,20^ using the Insight Toolkit (www.itk.org).

Briefly, the image was thresholded using a conservative threshold to provide initial seed points for a threshold level-set filter which provided a robust initial segmentation. Further sequential use of geodesic and Laplacian level-set segmentation filters provided refinement of the segmentation. Finally, slight manual adjustment was performed to remove any remaining artifacts.

**FIG. 1.**
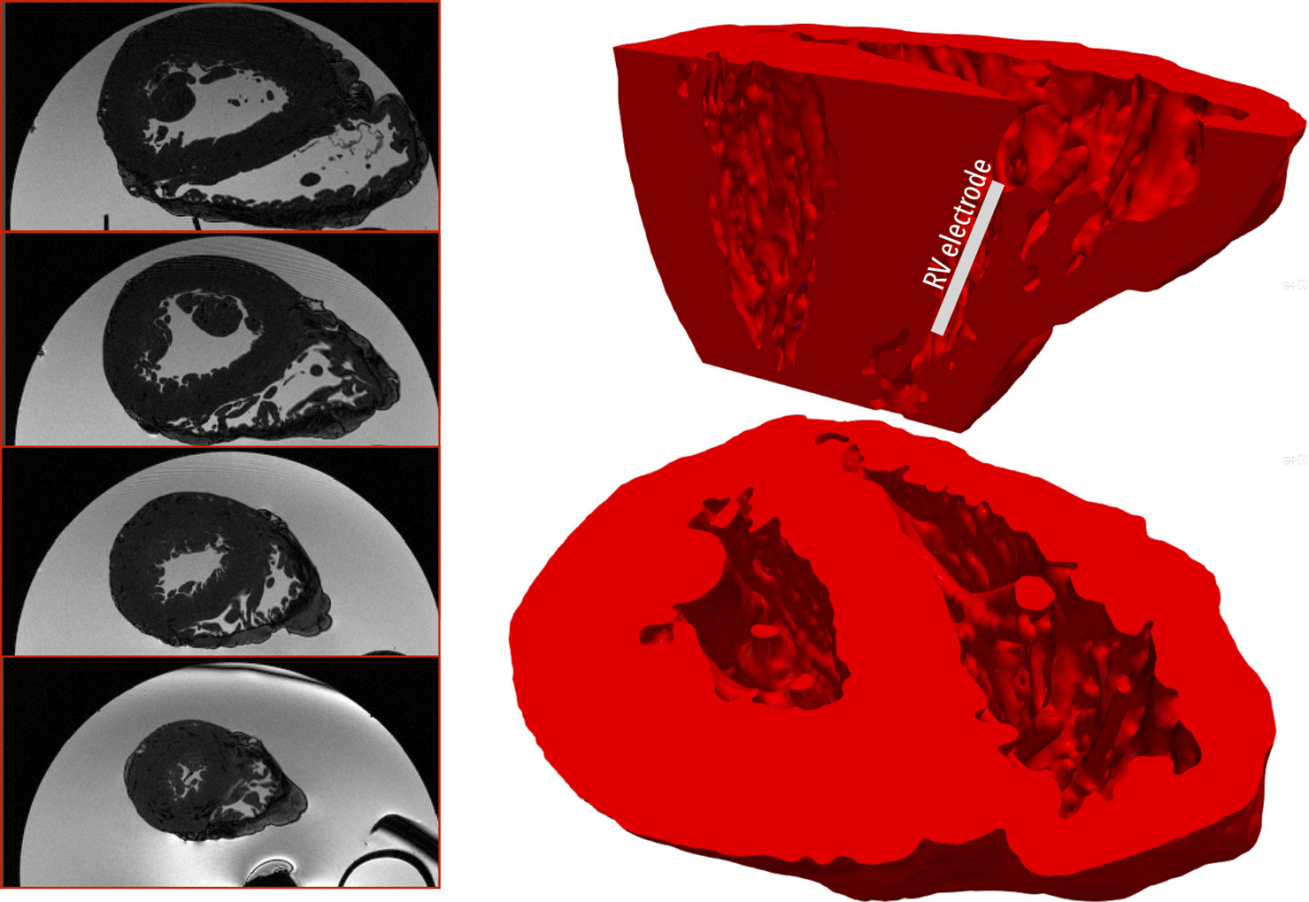
(Left) Human *ex-vivo* MR data shown through 4 different axial planes; (right) computational finite element model derived directly from MR data shown viewed from above (lower image) and with a sagittal clipping plane (upper image), demonstrating a high level of endocardial structural details present in the model.

The mesh generation software Tarantula was used to generate a tetrahedral finite-element mesh directly from the segmented voxel image stack.^21^ Tarantula uses an Octree-based algorithm that builds unstructured, boundary fitted, conformal finite-element meshes. Local refinement around tissue surfaces allows for accurate representation of the intricate cardiac microstructure. Full details of the underlying approach can be found in Ref. 21. The total mesh consisted of myocardial tissue, plus surrounding bath volume, constituting some 15.5 × 10^6^ nodes defining 92 × 10^6^ tetrahedral elements with a mean myocardial tissue edge length of 252 *μ*m. Figure 1 shows the *ex-vivo* MR data (left) and the generated computational finite element model (right).

#### 3. Fibre orientation definition

In the absence of DT-MRI or histology data for this preparation, a rule-based method was used to assign realistic fibre orientation, as used in previous studies.^11,12,22^ Briefly, local transmural and apico-basal directions were defined within each element of the mesh by computing the gradients from smooth potential fields satisfying Laplace's equation with different Dirichlet boundary conditions. Different combinations of positive/ground electrodes defined by the left ventricle (LV)/RV endocardial and epicardium (to obtain the local transmural direction) and the apex and basal planes (to obtain the local apico-basal direction) were used. Performing the cross-product of the local transmural and apico-basal directions provided a default circumferential vector. This default vector was then inclined by a transmural inclination angle ±60° from endo- to epicardium, based on the normalised transmural distance of the centroid of the element. Detached trabeculations had fibre vectors assigned to them along the axis of the structure.

### B. Simulating electrophysiological dynamics

#### 1. Governing equations

Electrical activation throughout the models was simulated based on the bidomain equations^23^

$$\nabla \cdot \sigma_{i}\nabla \phi_{i}=\beta I_{m},$$ (1)

$$\nabla \cdot \sigma_{e}\nabla \phi_{e}=-\beta I_{m}-I_{ei},$$ (2)

$$I_{m}=C_{m}\frac{\partial V_{m}}{\partial t}+I_{ion}(V_{m},\eta )-I_{s},$$ (3)

$$\nabla \cdot \sigma_{b}\nabla \phi_{e}=-I_{eb},$$ (4)

where *ϕ_i_* and *ϕ_e_* are the intracellular and extracellular potentials, respectively, *V_m_* = *ϕ_i_* − *ϕ_e_* is the transmembrane voltage, $\sigma_{i}$ and $\sigma_{e}$ are the intracellular and extracellular conductivity tensors, respectively, *β* is the membrane surface to volume ratio, *I_m_* is the transmembrane current density, *I_ei_* and *I_eb_* are extracellular stimuli applied in the interstitial space or the bath, respectively, *I_s_* is a transmembrane stimulus, *C_m_* is the membrane capacitance per unit area, and *I_ion_* is the membrane ionic current density which depends on *V_m_* and a set of state variables η. At tissue boundaries, no flux boundary conditions are imposed for *ϕ_i_*, with *ϕ_e_* being continuous at the interface. At the boundaries of the conductive bath surrounding the tissue, no flux boundary conditions for *ϕ_e_* are imposed.

In certain scenarios, the monodomain representation was used whereby the cardiac tissue is represented as a single conducting domain and the bidomain equations reduce to the monodomain equation, with conductivity given by the harmonic mean conductivity tensor or the effective bulk conductivity ($\sigma_{m}$).^24^

#### 2. Tissue electrophysiological properties

Cell membrane dynamics within the myocardial tissue were represented by a human ventricular ionic cell model.^25^ To reproduce the asymmetry of the membrane response to strong shocks delivered during the plateau phase of the action potential, the cell model was further augmented with two additional currents, an electroporation current and a hypothetical potassium current that activates at larger positive polarizations beyond +160 mV, as used in previous studies.^26^

Conductivities along the fibre and cross-fibre directions were based on previous experimentally derived values^27^ within the intracellular (0.174 S/m, 0.0193 S/m) and extracellular (0.625 S/m, 0.236 S/m) domains, respectively. These were then uniformly scaled to reduce conduction velocity by 25%, in line with similar reductions shown during heart failure, to account for the experimentally observed lateralization and hypophosphorylation of C×43 during heart failure which controls the flow of current between cells.^28,29^ Conductivity of the surrounding extracellular bath was set to 1.0 S/m (isotropic).^30^

#### 3. Computational considerations

The bidomain equations were solved with the Cardiac Arrhythmia Research Package (CARP).^31^ The specifics of the numerical regimes used in CARP have been described extensively elsewhere.^31,32^ An ordinary differential equation time-step of 20 *μ*s was used during pre-pacing and ventricular tachycardia (VT) induction simulations, with a refined time-step of 5 *μ*s used during all bidomain shock delivery simulations. Visualisation of results was performed with the custom written Meshalyzer software (courtesy of Dr Edward Vigmond) and the Paraview software (www.paraview.org/).

### C. Stimulation protocol

#### 1. Pacing

In order to initially assess the effects of applied shocks on structural heterogeneity alone, in the absence of significant heterogeneity in refractoriness, the entire tissue was uniformly stimulated by a transmembrane stimulus of strength 0.003 *μ*A cm^−3^, 2 ms duration applied to all nodes in the model. The model was paced 5 times at a basic cycle length of 500 ms in this manner in which the monodomain approximation was used. The state of the tissue was then check-pointed at different coupling intervals (CIs) following the 5th S1 stimulus between 240 ms (relatively refractory) to 340 ms (recovered).

#### 2. Shock application

Single monophasic shocks were applied to the tissue between an electrode located in the RV cavity and an external ground, outside the ventricles in order to approximately replicate an ICD electrode setup and to ensure a transmural field. Specifically, the RV electrode was 4 cm in length and 400 *μ*m in diameter, placed approximately in the centre of the RV cavity, aligned with the apex-base axis, as shown schematically in Fig. 1. The ground electrode was defined to be all 4 walls of the bounding box exterior to the ventricles in the horizontal plane. Shocks were applied of varying strength of 1, 3, 5, and 10 V, for a duration of 10 ms. Initially, anodal shocks were applied with the RV electrode being the positive electrode. In the latter part of the study, cathodal shocks were also applied, with the RV electrode being the negative electrode.

#### 3. Field strength and shock energy

Field strength (in V/cm) was heterogeneous within the model, being stronger near the RV electrode and decaying away from it. To give an indication, approximately 2% of the tissue experienced a field strength >5 V/cm during an anodal 10 V shock (assuming a piecewise-constant field for each element volume). Thus, all shocks considered here were significantly below conventional defibrillation thresholds which typically require >95% of the tissue to have field-strengths >5 V/cm.^33,34^

To further relate to specific low voltage protocols, shock energy was computed by

$$\text{Energy}=\frac{1}{R}\int_{0}^{T}V{\left(t\right)}^{2}dt,$$ (5)

where *T* is the duration of the shock, *V* the applied voltage, and *R* the resistance of the set-up (assumed to be constant). The resistance was computed by first finding the total current passing into the ground electrode during a fixed (known) applied voltage using

$$\int_{S}\sigma_{b}\nabla \phi_{e}\cdot \hat{n}dS,$$ (6)

where *σ_b_* is the conductivity of the bath (a scalar), ∇*ϕ_e_* is the gradient of the extra-cellular potential, and $\hat{n}$ is the normal to the surface of the ground electrode. The surface *S* represents the entire surface of the ground electrode. Using this method, the resistance of the electrode setup was found to be 36.4 Ω which was seen to be approximately constant within our range of applied voltages. This resistance gave computed shock energies of 0.0003 J, 0.002 J, 0.007 J, and 0.027 J for shocks of 1, 3, 5, and 10 V, respectively, which are all within the low-energy protocol regime.^2,5,6^

#### 4. VT induction

In addition, a sustained episode of VT was induced using a prescribed stimulus to the refractory tail following an apically paced beat (again following 5 preconditioning S1 beats). Such a protocol successfully initiated a sustained episode of VT, characterised by 2 main large reentrant circuits with transmural filaments. As with pacing preconditioning, the monodomain representation was used for VT simulations prior to shock delivery.

### D. Data analysis

Tissue was deemed to be activated for nodal *V_m_* values > −20 mV. Analysis of percentage of tissue activated was performed on the entire endocardial surface (RV and LV) in addition to the total myocardial volume at shock-end and 10 ms post-shock which allowed analysis of the initial effects of wavefront propagation initiated from the shock.

During episodes of VT, the complexity of the arrhythmia was quantified by analysis of filaments within the myocardium. Filaments were defined using the intersection of the *V_m_* isosurface at  −20 mV and $\frac{dV_{m}}{dt}=0$ method with the implementation described previously in Ref. 35. The total number of individual filaments at each instance in time was summed.

In the remainder of this manuscript, we refer to positive electrodes as “anodes” and negative electrodes as “cathodes,” with potential differences with respect to ground or zero-potential electrodes.

## III. RESULTS

### A. Excitation mediated by VEs from detached trabeculae

**FIG. 2.**
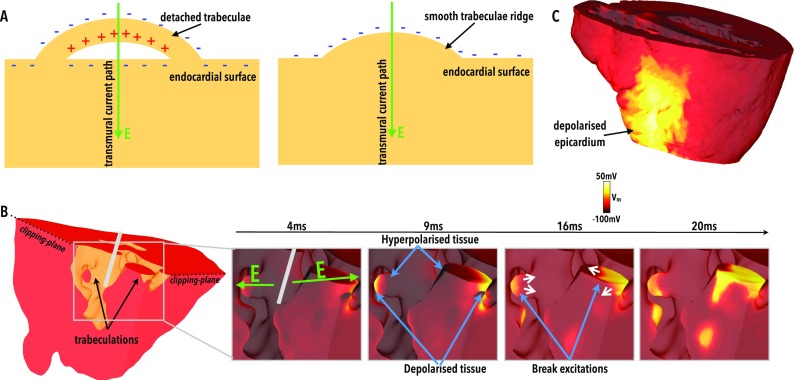
Depolarised VEs due to detached trabeculation. (a) Schematic diagram showing an example of a detached (left) and attached (right) trabeculae along with the resulting VE formation due to current movement. (b) Highlighted region of the RV endocardial surface (left) showing two detached trabeculations. 4, 9, 16, and 20 ms panels (right) demonstrate the evolution of *V_m_* distributions at these respective times following a shock of SS 5 V, CI 280 ms (relatively refractory tissue). Green arrows in 4 ms panel show the direction of electric field, with the location of the catheter schematically highlighted in grey. (c) Epicardial view of the same shock as in panel B showing depolarised regions of the epicardial surface.

Application of anodal shocks from the RV electrode was seen to produce regions of *depolarisation* located on the distal side of those trabeculations which become entirely detached from the endocardial surface, relative to the electrode. An example of such a detached trabeculae is shown schematically in Fig. 2(a) (left), along with the resulting de- and hyperpolarisation caused by a transmural current path through the tissue. Note that a trabeculae that does not become detached from the surface would appear as a characteristic “trabeculae ridge” along the endocardium. A smooth endocardial surface would show hyperpolarisation only, with no regions of depolarisation, as shown in Fig. 2(a) (right). These trabeculae depolarisations are highlighted in Fig. 2(b) which shows a small portion of the ventricular model, highlighting two separate trabeculations (left), both fully detached from the endocardial surface. The subsequent depolarisation caused by the anodal shock to parts of the *epicardial* surface is also shown in Fig. 2(c). More appreciable depolarisation is seen for stronger shocks and at longer CIs. The 4 and 9 ms panels of Fig. 2(b) show that the proximal side of the trabeculation (relative to the electrode) is hyperpolarised as current enters the myocardium here first. Depolarisation then occurs where current *leaves* the myocardium on the distal side of the trabeculation, prior to re-entering the myocardium at the endocardial surface where it causes further hyperpolarisation. As the initial state of the tissue at shock application was relatively refractory, propagation does not initially occur from these isolated regions of depolarisation, as the surrounding tissue is not excitable. However, the hyperpolarising action of the shock causes these regions to regain their excitability; thus, upon shock cessation (10 ms), break excitations occur from the depolarised regions into the hyperpolarised regions on the opposite side of the trabeculations. The result is local capture of tissue and isolated wavefront initiation from the endocardial surface which would otherwise not be present without this shadowing effect of the detached trabeculations.

**FIG. 3.**
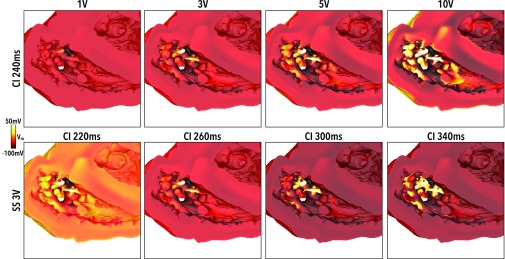
Effect of shocks on the endocardial surface looking into RV. Shock-end *V_m_* distributions for (upper panels) a fixed initial tissue state (CI 240 ms) as the SS increases from 1 to 10 V, and (lower panels) a fixed applied SS (3 V) as the initial tissue state varies between CI of 220 and 340 ms.

Figure 3 highlights this mechanism within the entire RV cavity, showing how the depolarised and hyperpolarised tissue presents changes as shock strength (SS) varies (for a fixed initial tissue state, upper panels of Fig. 3) and as the initial tissue state varies (for a fixed SS, lower panels of Fig. 3). As the SS increases (upper panels), increased amounts of both hyperpolarised and depolarised tissue are seen on the endocardial surface; the latter being present due to the presence of detached endocardial trabeculations. It is noted that only at the strongest SS of 10 V does the epicardial surface becomes appreciably depolarised by the action of the shock. However, due to the fact that this shock was applied at a relatively early CI (240 ms), the intramural tissue remains relatively refractory post-shock, preventing the propagation of excitation from this depolarised epicardial surface. The lower panels of Fig. 3 show that (with the exception of CI 220 ms), shocks applied at later CIs cause larger amounts of the endocardial RV surface to become depolarised. The mechanism describing the response of the tissue at CI 220 ms is described later.

### B. Endocardial make and break excitations

**FIG. 4.**
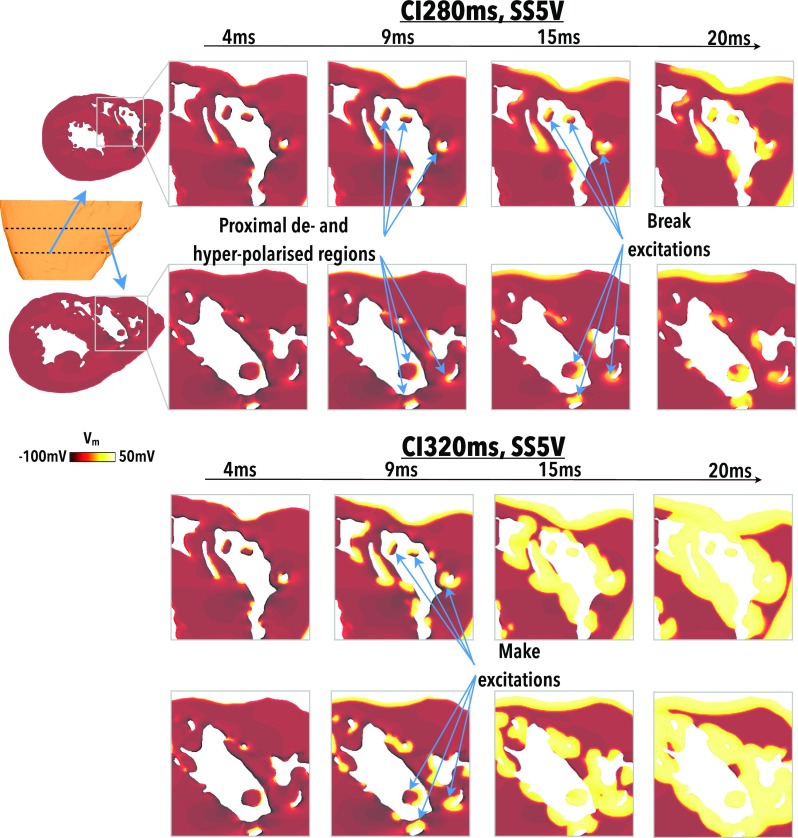
Elicitation of make and break excitations from anodal shocks. *V_m_* distributions at 4, 9, 15, and 20 ms following shock initiation (0 ms) for 5 V shocks applied to largely recovered (*CI*320 ms, upper panel) and largely refractory (*CI*280 ms, lower panel) tissue. Two different clipping planes are used to examine *V_m_* levels towards the apex (top rows) and towards the base (bottoms rows), as shown in the schematics.

The elicitation of make and break excitations via the VE mechanism mediated by detached trabeculae in Fig. 2 is now highlighted in more detail. Figure 4 compares intramural *V_m_* distributions within highlighted regions of the RV throughout the duration of, and up to 10 ms following, a shock of SS 5 V applied to tissue that is relatively refractory with CI 280 ms (upper panels) and tissue that is recovered with CI 320 ms (lower panels).

At both CIs, the action of the shock creates depolarisation along the epicardial surface and in regions of detached trabeculae. At CI 320 ms (lower panel), the tissue is recovered and the depolarising effect of the shock initiates make excitations (which propagate upon shock onset) which are free to propagate into the surrounding recovered tissue. Such excitations propagate from multiple locations on the endocardium, rapidly activating the whole endocardial surface. This effect is facilitated by the hyperpolarising effect of the shock on the rest of the endocardium, increasing the excitability of the endocardial surface and increasing conduction velocity. By 10 ms postshock (20 ms image), a largely contiguous wavefront is formed that propagates transmurally from endo- to epicardium. This wavefront meets the wavefront initiated from the epicardial surface, acting to rapidly close-down the intramural excitable tissue.

At CI 280 ms (upper panel), the tissue is still refractory at the time of the shock and immediate propagation from make excitations does not occur. Instead, in addition to regions of depolarisation formed at detached trabeculae, corresponding regions of hyperpolarised tissue form proximal to the anodal electrode, causing these regions to regain excitability. Such regions are sufficiently close to one-another that propagation from depolarised to hyperpolarised tissue occurs, in the form of break excitations (excitations which propagate upon shock termination). Because only previously hyperpolarised regions may be excited (as these were made excitable via hyperpolarisation), a smaller number of isolated wavefronts are initiated from the endocardial surface, in contrast to the large number of wavefronts in the *CI*320 ms case (lower panel) which rapidly formed a large contiguous wavefront.

**FIG. 5.**
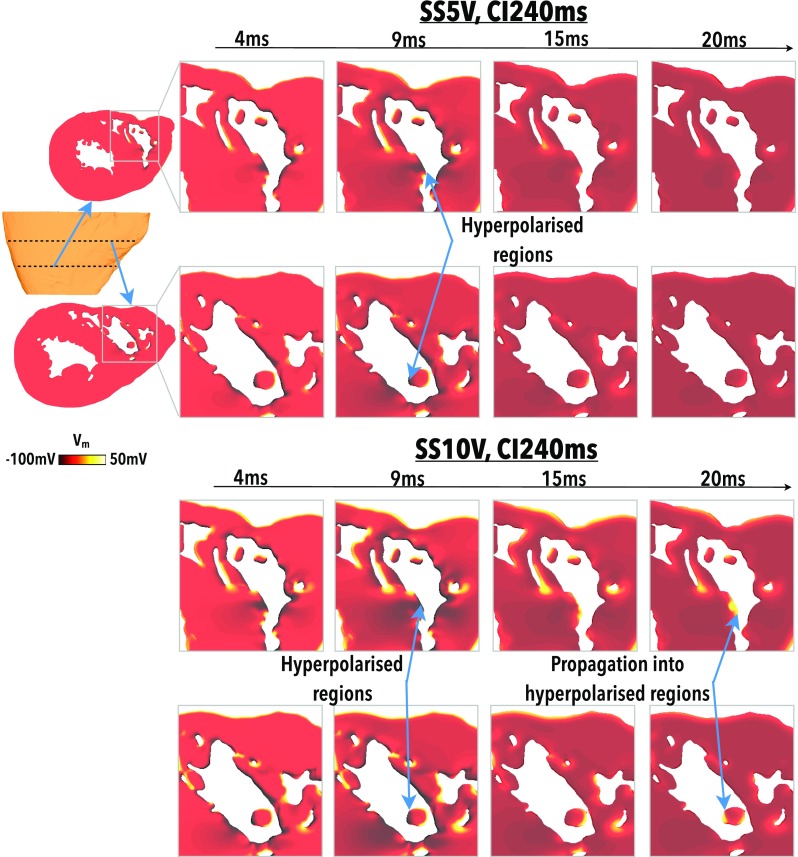
Failure/success of break excitations in refractory tissue. *V_m_* distributions at 4, 9, 15, and 20 ms following shock initiation (0 ms) for a 5 V (upper panel) and 10 V (lower panel) shock applied to largely refractory tissue (CI240 ms). Two different clipping planes are used to examine *V_m_* levels towards the apex (top rows) and towards the base (bottoms rows), as shown in the schematics.

Figure 5 shows a similar situation to Fig. 4, but for the case of a much earlier CI of 240 ms. As CI is decreased, regions of depolarisation on the endocardial surface are still produced; however, the higher initial *V_m_* levels at shock initiation mean that the hyperpolarising effect of the shock is less pronounced. Consequently, a 5 V shock applied to the tissue is not sufficiently strong to elicit break excitations into these more weakly polarised areas, which have not had their excitability fully restored, as shown in Fig. 5 (upper panel). However, if SS is increased, the hyperpolarising effect of the shock becomes stronger and these affected regions have their excitability restored such that they can provide a pathway for propagation from the depolarised regions, as shown in Fig. 5 (lower panel) in which a 10 V shock is applied.

### C. Activation of the endocardial surface dependent on state of refractoriness and applied shock strength

In Sec. II B in the sections above how detached trabeculae cause local regions of depolarisation which may they propagate out and capture neighbouring tissue via both make and break excitation mechanisms. Here, we quantitatively examine the dependence of these effects on both the strength of the applied shock and the state of refractoriness of the tissue. Note that here we do not quantify the effects of cathodal shocks, as this shock polarity is expected to cause widespread depolarisation to the endocardium surface; regions of detached trabeculae would merely induce isolated regions of hyperpolarisation (which do not propagate).

**FIG. 6.**
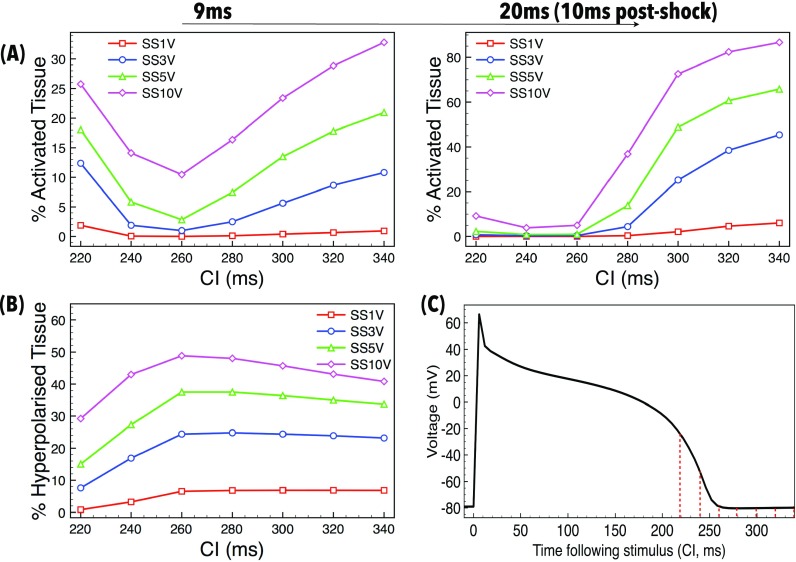
Quantification of endocardial activation as a function of applied SS and tissue refractoriness (CI). (a) Percentage of the endocardial surface activated (*V_m_* > −20 mV) by the shock 9 ms (left) and 20 ms (10 ms post-shock, right) after shock initiation as a function of CI for different applied SSs. (b) Percentage of the endocardial surface hyperpolarised (<−80 mV) by the shock 9 ms after shock initiation as a function of CI for different applied SSs. (c) Change in *V_m_* over time following preconditioning stimulus, showing the refractory state of tissue when each shock is applied (CI, shown by red dotted-lines).

Figure 6(a) shows the percentage of the RV endocardial surface tissue which is activated (with *V_m_* > −20 mV, as defined in the Methods) at 9 ms (shock-end, left) and 20 ms (10 ms postshock, right) as a function of CI for different applied SSs. Note that the maximum polarisation level present in the domain prior to the earliest shock application (CI of 220 ms) was −25 mV. Therefore, any tissue with polarisation level > −20 mV following the shock must have been directly positively affected by the shock itself. At the end of the shock, depolarised tissue exists on the endocardial surface (driven by the mechanism of detached trabeculae, described above) for all SSs and CIs considered, with higher SSs yielding higher percentages of tissue activated, as expected. For a given SS, at longer CIs, as the CI increases the amount of endocardial tissue activated also increases (as seen in Fig. 3). Here, make excitations occur; the more recovered the tissue (longer CI), the more readily the make excitations propagate during the stimulus itself, activating large regions of endocardium by shock-end (as shown in Fig. 4). Higher SSs activate more regions by the proposed mechanism, resulting in more sources of wavefronts which act to activate more of the endocardium. At shock-end, at the highest CI (340 ms) and strongest SS (10 V), a substantial proportion of the endocardium is activated, which rises rapidly to over 85% just 10 ms postshock. Importantly, however, even the weakest shocks (1 V, red line) applied at the longer CIs successfully result in small regions of the endocardium being activated, which subsequently propagate out capturing neighbouring tissue to cause approximately 10% to be activated by 10 ms postshock.

At shorter CIs (<260 ms), make excitations do not occur as the tissue is refractory upon shock application. However, regions of de- and hyperpolarisation are still produced due to the action of the shock, as shown in Figs. 4 and 5. During the shock, Fig. 6(a) (left) shows that the stronger the shock, the larger the amount of tissue is activated, as expected. Perhaps counter intuitively, as CI shortens, the percentage of endocardium activated increases. This effect occurs due to the fact that the tissue at the time of shock application is at a higher level of refractoriness [shown in Fig. 6(c)], meaning that it is easier to elevate *V_m_* levels to “activated” values; such a phenomena is related to the dip in the unipolar anodal strength interval curve.^36^ However, despite large regions of the endocardium being activated at shock-end at the lowest CIs, Fig. 6(a) (right) shows that these activated regions do not propagate rapidly (due to the large regions of surrounding refractory tissue, as highlighted in Figs. 4 and 5), causing relatively small proportions of the endocardium to be activated 10 ms postshock (<10%).

Figure 6(b) quantifies the amount of the endocardial surface that is hyperpolarised (<−80 mV) at the end of the shock. For all SSs, the amount of tissue that is hyperpolarised remains relatively constant for CIs above approximately 260 ms. As CIs shorten, however, the amount of hyperpolarised endocardial tissue decreases, as the tissue is at a progressively higher level of refractoriness pre-shock.

### D. Post-shock propagation from endocardial activations

In Secs. III A–C we have demonstrated how the presence of detached trabeculations within the human ventricle creates large regions of depolarised tissue across the endocardial surface after field stimulation. Here, we examine how these regions of depolarised tissue subsequently propagate out to capture the greater myocardial tissue volume.

**FIG. 7.**
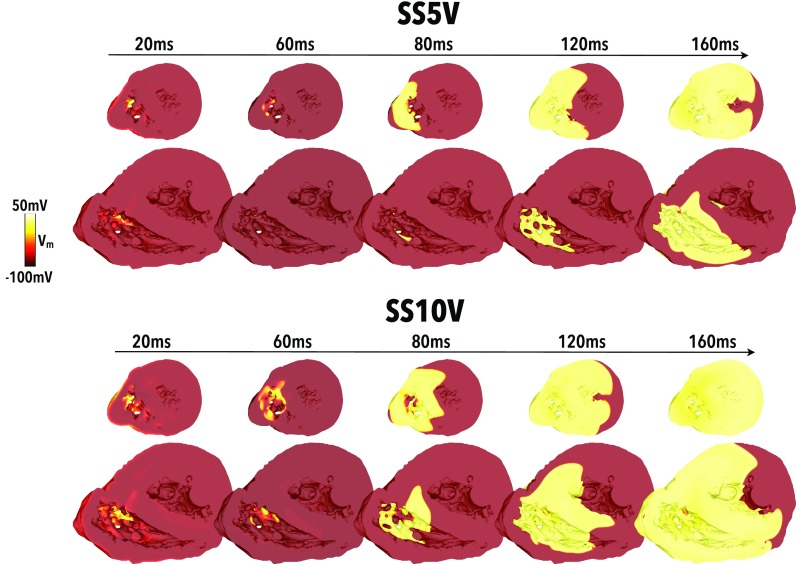
Initiation of post-shock propagation following capture of the endocardial surface. *V_m_* distributions shown at different times between 20 and 160 ms after shock onset for shocks of 5 V (top panel) and 10 V (lower panel) applied to tissue at a CI of 240 ms. Data are shown across two clipping planes taken from similar locations as in Fig. 4.

Figure 7 shows the evolution of post-shock activity up to 160 ms after the cessation of the shock for the case of a 5 V and a 10 V shock applied to tissue at a CI of 240 ms (relatively refractory). Weaker shocks did not have sufficient strength to activate tissue via make/break excitation mechanisms and post-shock activation rapidly died away. Importantly, it is noted that activation only initiates from the endocardial surface via the mechanism of proximal de- and hyperpolarised regions described in In Sec. III B and III C. Although the epicardial surface is slightly depolarised by the shock, activation does not propagate from here as the surrounding tissue remains refractory. Comparing the two SSs in Fig. 7, we see that the stronger shock depolarises a larger region of endocardial tissue proximal to the electrode. The 10 V shock also causes a stronger region of hyperpolarisation to be formed (more clearly evident in the images of Fig. 5 which show the same applied SS-CI as here). Consequently, the surrounding myocardial tissue is captured much more rapidly in the case of the 10 V shock, compared to the 5 V shock, with the entire RV being captured after approximately 140 ms.

### E. Comparison of the total myocardial volume activated by anodal vs cathodal shocks

**FIG. 8.**
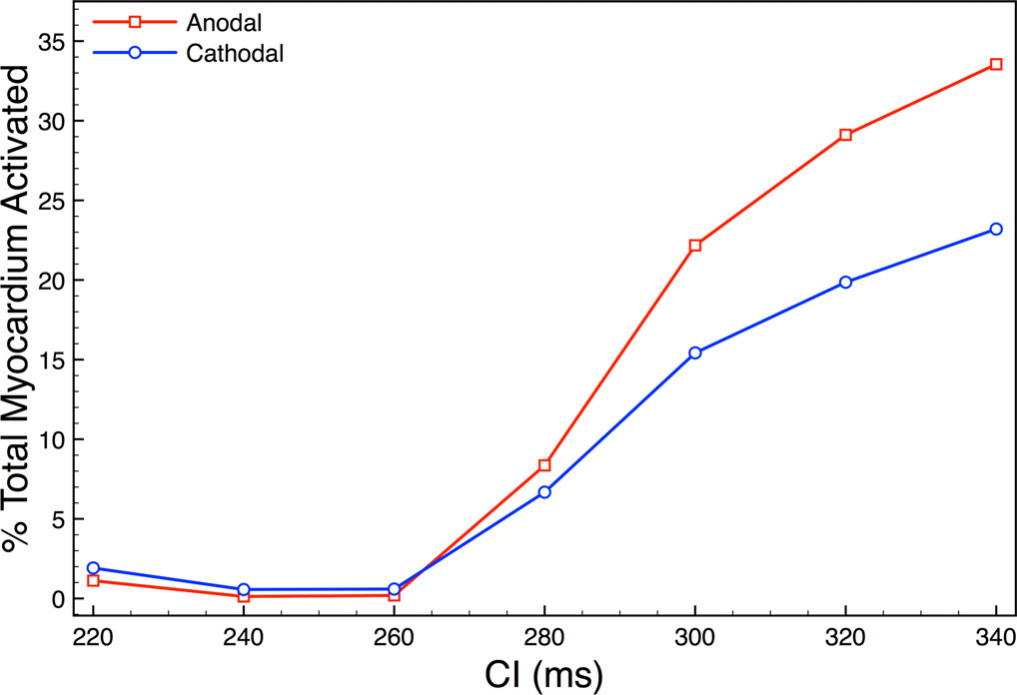
Total myocardial volume activated 20 ms after shock onset (10 ms after shock cessation) following either 10 V anodal (positive, red line) or 10 V cathodal (negative, blue line) shocks as a function of state of tissue refractoriness (CI) at the time of shock application.

Figure 8 quantitatively evaluates the proportion of the entire myocardium which is activated 20 ms after shock initiation (10 ms postshock), as the timing of the shock (CI) is varied for a fixed (stronger) SS of 10 V. Here, we also directly compare the effects of anodal (red line) and cathodal (blue line) shocks.

As can be seen in the figure, either shock polarity struggles to activate an appreciable proportion of the myocardial volume 10 ms following shock cessation for shorter CIs (≤260 ms). As shown previously in Figs. 3 and 5, positive (anodal) 10 V shocks applied to tissue in such a state of refractoriness do not depolarise significant regions of the epicardial surface, as the field is relatively weaker. Post-shock, activations are only initiated from isolated regions on the endocardial surface (via the mechanism proposed in Fig. 2). At short CIs, these regions only propagate into hyperpolarised “channels” of tissue in which excitability has been restored (Fig. 5, lower). On the other-hand, cathodal (negative) shocks would not be expected to depolarise the (smooth) epicardial surface at all, as here current only enters the myocardial causing the epicardium to become hyperpolarised. Instead, they would only be expected to cause widespread activation of the endocardial surface, except in distal regions of detached trabeculae where hyperpolarisation would occur (in a similar, but opposite, mechanism to that proposed in Fig. 2). As the electric field is approximately radial, it is much weaker on the epicardial surface than the endocardial surface (close to the electrode). For cathodal shocks, as the field is stronger on the endocardium, relatively more depolarisation occurs on the endocardium, compared to that on the epicardium with anodal shocks. However, as described above, anodal shocks still depolarise small regions of the endocardium via the proposed mechanism. Thus, both anodal and cathodal shocks activate the endocardium, but the latter is significantly more widespread, explaining the slightly higher proportion of the total myocardium activated by cathodal shocks for shorter CIs in Fig. 8.

However, as CI increases and the tissue becomes less refractory upon shock application, Fig. 8 clearly shows that the anodal shocks cause a much higher proportion of the myocardium to be activated 10 ms after shock cessation. In such cases, the positive shocks can successfully activate propagating wavefronts from the epicardial surface, something the negative shocks never do, regardless of the CIs. In addition, small regions of depolarised tissue on the endocardium become more widespread and propagate as make excitations, as shown in Fig. 4. The combination of excitation wavefronts being initiated from both epi- and endocardial surfaces results, in the case of anodal shocks, in a more rapid activation of the total myocardial volume compared to cathodal shocks at longer CIs.

### F. Effect of anodal and cathodal shocks applied to VT

**FIG. 9.**
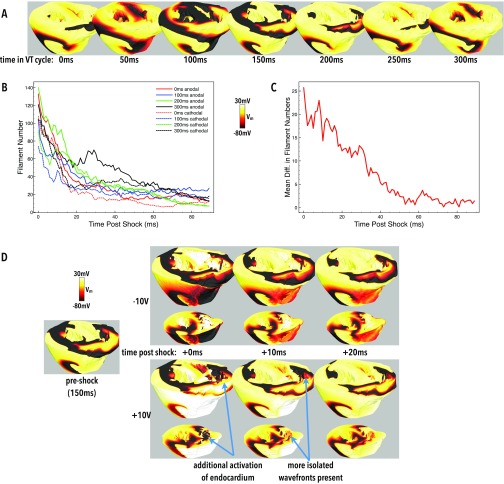
Post-shock response during episodes of sustained VT. (a) Snapshots of *V_m_* distributions at different relative stages of the VT cycle. (b) Plots of filament counts in the first 90 ms following both anodal (solid lines) and cathodal (dashed-lines) shocks applied to the initial VT states in (a). Matched colours correspond to the same pre-shock state. Only data for shocks applied to pre-shock states of 0, 100, 200, and 300 ms are shown for clarity. (c) Mean paired differences (anodal minus cathodal) of filament counts in the first 90 ms after shock-end, averaged over all initial states. (d) Snapshots of *V_m_* distributions in the 40 ms following a 10 V cathodal (top images) and 10 V anodal (bottom images) shock applied to tissue at a relative time of 150 ms of the VT cycle [panel (a)]. The monodomain representation was used for VT simulation prior to shock delivery.

Having examined the differences between anodal and cathodal shocks applied to tissue in a uniform state of refractoriness above, we now investigate how such fundamental differences between these applied shock polarities interact with tissue in more heterogeneous states of refractoriness during an arrhythmia. Both anodal and cathodal 10 V shocks were separately applied to tissue at 7 different instances in time, separated by 50 ms, during a simulated episode of VT, as described in Sec. II C 4. The initial tissue states prior to shock delivery are shown in Fig. 9(a). Figure 9(b) then seeks to quantify the interaction of the applied shocks with the tissue by plotting how the number of filaments is altered in the post-shock period. As shown in the figure, none of the shocks successfully terminated VT, as may be expected by applying a single, relatively weak monophasic shock. In fact, all shocks induce the formation of more filaments compared to those in existence pre-shock (although these additional filaments die-away with time). Such post-shock filaments arise due to the formation of small propagating wavefronts from VE sources within these structurally heterogeneous models. Comparing shock polarities, we can clearly see that the anodal shocks tend to generate more filaments post-shock than cathodal shocks. This is also confirmed in Fig. 9(c) which performs a pair-wise comparison, comparing the mean difference in post-shock anodal versus cathodal filaments at all instances post-shock.

This additional number of filaments is driven by the extra wavefronts initiated from endocardial structures during anodal shocks, as highlighted in Figs. 3, 4, 5, and 7. Some evidence for this increased number of induced wavefronts can be seen in Fig. 9(d) which shows the tissue state 0–40 ms post-shock. The additional activation of the endocardium in the RV can be seen for the anodal shock, which generates wavefronts which then activate more tissue in the instances after the shock, as suggested by the findings in Sec. III D.

## IV. DISCUSSION

The interactions of monophasic shocks applied via implanted electrodes within the human ventricle are important to understand with respect to optimising recent promising experimental low-energy defibrillation protocols,^2–7^ facilitating their translation into the clinic. In this study, we used a human bi-ventricular model, containing unprecedented levels of anatomical detail, to simulate the interaction of monophasic shocks with fine-scale endocardial structures. Our findings suggest that the interaction of anodal shocks with the highly trabeculated nature of the human endocardium drives important differences in post-shock responses, relative to cathodal shocks. At short CIs, we uncover the mechanism responsible for these differences as being primarily due to break excitations from strongly de- and hyperpolarised regions in close proximity within detached trabeculae structures, which are particularly numerous in the human RV. At longer CIs, make excitations from depolarised regions of these detached structures also produce important differences.

### A. Explanation of asymmetry in response to anodal/cathodal shocks

Conventional strong defibrillation shocks applied from ICDs have biphasic truncated exponential waveforms, with the first phase having a stronger leading-edge than the second. Empirical clinical evidence strongly indicates a superiority of shocks with an anodal first phase.^37^ A number of different theories have been suggested for this, including charge-burping^37^ and cellular ionic responses.^38^

Monophasic shocks, as used in both low and high energy experimental studies, are more simple, square waves. As current must pass through an even number of external surfaces between electrodes, it may be expected that monophasic shocks have symmetrical effects for anodal/cathodal polarities. Experimental observations and bidomain theory suggest that a cathode placed in the RV should have a depolarising effect on the RV endocardium (as current leaves the intracellular domain to reach the cathode) and a hyperpolarising effect on the RV epicardium (as current enters the intracellular domain). Conversely, an anode in the RV should have a depolarising effect on the *epicardium* and a hyperpolarising effect on the *endocardium*, producing a similar overall effect to cathodal shocks. This is indeed what has been shown in previous modelling studies using relatively anatomically simple ventricular geometries^39,40^ with smooth surfaces.

In this study, we showed that the anatomically complex nature of the endocardium induces an asymmetry in the response of anodal and cathodal shocks. Detached trabeculations provide an additional set of surfaces with which current must pass through when travelling between electrodes. As shown in Fig. 2, as current enters the proximal side of the trabeculation (from an anodal RV catheter), hyperpolarisation occurs at the tissue surface; however, when it leaves the trabeculation on the distal side, depolarisation of the tissue occurs. Consequently, regions of depolarisation are present on the endocardium which would not otherwise occur on a smooth endocardial surface. If the pre-shock state of the tissue is recovered, then these excitations may initially begin to propagate as make excitations, as shown in Fig. 4, helping to rapidly activate the myocardium.

### B. Activation of refractory tissue and relation to shock strength

**FIG. 10.**
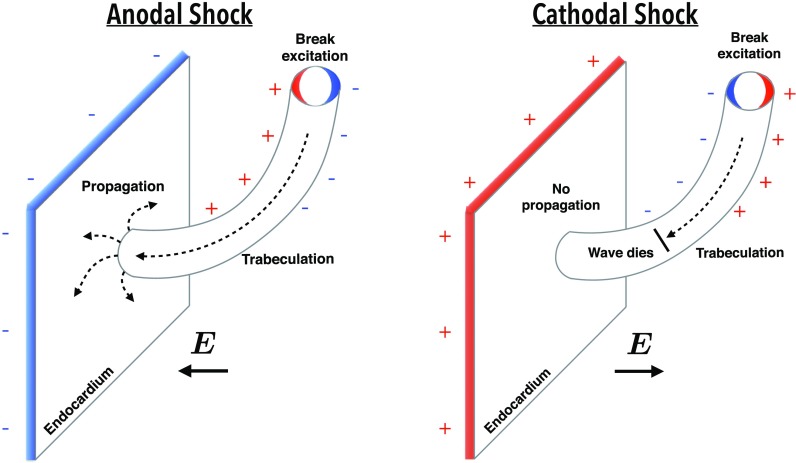
Schematic representation of the effect of an anodal (left) and cathodal (right) shock on a region of the endocardial surface close to a detached trabeculation. Both shock polarities show the initiation of propagation from the proximal de- and hyperpolarised regions within the detached trabeculation, however, only the anodal shock facilitates capture of the smooth endocardial surface due to hyperpolarising effect of the positive shock.

Such a mechanism may also facilitate propagation in pre-shock refractory tissue. Due to the relatively smaller width of a trabeculation, these regions of depolarisation are inherently proximal to regions of hyperpolarisation, where any pre-shock refractoriness may have been restored. Thus, post-shock, activation rapidly spreads from the depolarised areas into the hyperpolarised regions, which may then propagate and activate the rest of the endocardium which is hyperpolarised, as shown in Fig. 5. Figure 10 shows this process schematically, demonstrating how anodal shocks may initiate isolated wavefronts that propagate from detached trabeculations, capturing the endocardial surface. A key feature of this process is that the excitability of the smooth endocardium itself has been restored by the shock, providing a post-shock avenue for propagation. In contrast, in the case of cathodal shocks, although a similar mechanism of initiation of wavefront propagation from the detached trabeculation occurs, propagation is blocked at the endocardium, due to the depolarising action of the cathodal stimulus on the smooth endocardial surface.

Overall, the mechanisms of propagation between de- and hyperpolarised regions within detached trabeculations are similar to the VE-driven anodal/cathodal make/break excitations under unipolar stimulation.^36^ Note that such a mechanism is also present in papillary muscles, but due to their larger size, the de- and hyperpolarised regions are further apart, and so post-shock activation does not occur so readily.

An important consequence of this finding is that, depending on the exact state of tissue refractoriness, activation may be initiated on the endocardium near the RV electrode during an anodal shock, *before* it occurs on the epicardium; although depolarised, the epicardium is *not* proximal to a region of hyperpolarisation to restore excitability and provide an excitable pathway for it to propagate. This finding is particularly important for consideration in low-energy defibrillation protocols, where field strengths may be insufficient for activating epicardial surfaces (with anodal shocks). However, even with higher field strengths (capable of activating the epicardium), our results suggest that both activation from (RV) epicardial and endocardial surfaces will occur, more rapidly shutting-down intramural excitable gaps, a mechanism uncovered from modelling studies as an important mechanism of biphasic defibrillation.^12^

### C. Electrode placement

The strength of the induced VEs within the trabeculations needs to be sufficient so as to elicit excitations, and thus, this effect depends on local field strength. Proximal to the electrode, the field is strongest due to the geometric scaling effect. Thus, those trabeculations closest to the RV electrode will experience the strongest VE effects. Indeed, the basal region of the RV, where the electrode is most usually placed in an ICD, is the most highly trabeculated, which explains the relatively widespread occurrence of induced activations in the setup analysed here, as shown in Fig. 3. Thus, alternative electrode placements, for example, plate electrodes as used typically in Langendorff preparations^39,40^ or sub-cutaneous ICDs which do not use an electrode in the ventricular cavities may not experience such a mechanism. Furthermore, in the less trabeculated LV, although present, this mechanism is much weaker. Our findings suggest that future optimisation of these protocols should involve leveraging this mechanism within the LV as well as the RV.

### D. Species dependence

Most recently published low-energy defibrillation protocols have used anodal monophasic shocks, which may be due to noticed increases in efficacy over cathodal shocks driven by the interactions with endocardial structures suggested here. A very important consideration, however, is the differences in species used in these studies, specifically related to the anatomical complexity of their endocardial structures. As the underlying mechanisms suggested here are entirely due to the trabeculated structure of the endocardium, the degree of trabeculation of a particular species may make these effects more or less apparent. In species with relatively smooth endocardial surfaces, such as rabbit and pig, with few detached trabeculations, we thus suggest that the differences between anodal/cathodal shocks will be less apparent than in humans, which is known to have a highly trabeculated endocardium. The interaction of endocardial structures presented here should thus be kept in mind when interpreting shock effects in different species.

### E. Anti-arrhythmia effects of monophasic shocks

Recently published low-energy defibrillation protocols have used very specific (and different) shock protocols, and have been conducted on different species. In this work, we did not try to replicate these low-energy protocols, but instead sought to understand better the effects of monophasic shocks on the human anatomy.

We demonstrated how monophasic shocks of different polarities may have significantly different effects on the dynamics of the arrhythmia, with anodal shocks inducing more post-shock filaments than cathodal. These differences may be attributed to anodal shocks creating more post-shock activations originating from trabeculation structures, primarily in the RV, than cathodal shocks, as uncovered in Secs. III A–C. Although the creation of large, single filaments post-shock is known to be arrhythmogenic (re-inducing arrhythmias post-shock via the shock-induced phase-singularity mechanism^41^), we suggest that large numbers of small isolated filaments may be anti-arrhythmic.

The ultimate goal of defibrillation is to shut-down excitable gaps, activating the bulk myocardium. Here, we have used filament counts as a surrogate for isolated wavefronts (induced from VEs). Thus, we suggest that the increased number of wavefronts initiated from anodal monophasic shocks facilitates defibrillation by more successfully removing excitable gaps, as these individual wavefronts ultimately annihilate with one another. Such conjecture is backed-up by the analysis in Fig. 8 which underscores how anodal shocks more rapidly activate the bulk myocardium than cathodal shocks. Ultimately, the increased defibrillation success of anodal monophasic shocks must be tested by a large, specific modelling or experimental study.

### F. Study limitations

Our human ventricular model did not include the presence of coronary vessels, as they were not present with sufficient clarity within the MR data to allow accurate segmentation. Vessels have been shown to be an important source of VEs for low-energy defibrillation protocols^6^ (as well as standard energy protocols^12^) However, bidomain simulations and theory suggest that activation occurs preferentially from endocardial invaginations before vessels^2^ at lower field strengths.^16^ The lack of vessels in our model has allowed us to dissect the specific effects of the trabeculation structure on monophasic shocks, in the absence of vessels. It is known that vessels would largely have a symmetrical response to changes in shock polarity.^42^

We would like to highlight that this study was performed on a single heart model and thus, our findings should be taken in this context. Although this sample was a representative healthy heart, anatomical variation between individuals, as well as likely structural changes during pathology, may well indeed affect our quantitative findings. However, the fundamental mechanism highlighted in this work (of current passing through the distal side of a detached endocardial structure during an anodal shock causing an isolated depolarising effect) will hold true regardless of patient specific changes in anatomy, although the density and specific location of these detached structures may well influence its overall effect on defibrillation.

There was some distortion to the structure of the RV due to the preparation of the sample for MR scanning, being slightly collapsed, whereas the LV maintained a reasonable systolic structure. This may have slightly affected the proximity of the RV electrode to the endocardial surface. However, due to the relatively high conductivity of the bath within the cavity, the majority of the voltage is “dropped” within the tissue and thus, the electrode-surface distance is thought to be less of a significant factor. Furthermore, in a fully contracting heart, the distance of the RV electrode to the tissue surface would be changing constantly as the RV contracts.

Finally, although the geometry and location of the RV electrode was representative of clinically used defibrillator electrodes, the ground electrode was not placed in a specific location outside the heart due to the absence of a torso. Instead, the surrounding edges of the bounding box were used as the ground in order to setup a shock vector that was radially outwards from the RV electrode, thus producing a transmural field at all parts of the myocardium. This allowed us to fully analyse the effect of current passing through detached trabeculae structures within all parts of both the RV and LV. Furthermore, it should be noted that, in the clinical scenario, the energy required to create a desired myocardial field-strength will be larger than the energies we quote in this work, due to the much larger domain (torso) and corresponding distances between electrodes.

## V. CONCLUSIONS

We have demonstrated that monophasic shocks of anodal polarity, applied from implanted electrodes, are more successful in activating the bulk myocardium compared to cathodal shocks. These differences are driven by the formation of VEs across trabeculations detached from the endocardial surface, which in-turn may initiate propagating wavefronts via make or break excitations, rapidly activating the rest of the endocardial surface which was previously hyperpolarised by the shock. This effect is not apparent following cathodal shocks as the endocardium is depolarised directly by the shock. Thus, following an anodal shock of sufficient strength *both* epicardium and endocardium may be activated, as opposed to just the endocardium following a cathodal shock; the relatively more smooth geometry of the epicardium prevents a symmetrical effect from occurring. This asymmetry in response to shocks of different polarity is thought to be especially apparent in humans relative to other species, due the highly trabeculated nature of the human endocardium—particularly in the RV where the implanted ICD electrode is often placed. When applied to episodes of sustained arrhythmias, anodal shocks induced more small, isolated propagating wavefronts which increased filament counts during the shock. Whether these effects are pro- or anti-arrhythmic may depend on the exact nature of the preceding arrhythmia and the exact protocols in which the shock is applied and thus requires further investigation. We suggest that these differences may be important to consider when optimising monophasic low-energy defibrillation protocols in different species.
